# Role of Ginger in management of nausea among patients receiving chemotherapy

**DOI:** 10.12669/pjms.40.9.8739

**Published:** 2024-10

**Authors:** Zamin Abbas Syed, Ammad Fahim, Mahpara Safdar, Rafia Imtiaz

**Affiliations:** 1 Zamin Abbas Syed, Principal (Nursing and Midwifery), Health Services Academy, Islamabad, Pakistan; 2 Ammad Fahim, Director ORIC, Indus Hopsital Health Network, Karachi, Pakistan; 3 Mahpara Safdar, PhD Human Nutrition, Assistant Professor at Department of Nutritional, Sciences and Environmental Design, Allama Iqbal Open University, Islamabad, Pakistan; 4 Rafia Imtiaz, Lecturer College of Physical, Therapy at Government College University Faisalabad, Faisalabad, Pakistan

**Keywords:** Chemotherapy, Ginger, Nausea, Vomiting, Emesis

## Abstract

**Objective:**

Cancer patients treated with chemotherapy often face variety of side effects, with nausea and vomiting being the most frequent. Ginger (*Zingiber officinale*), contains natural compounds that can speed up the metabolism and increase intestinal motility. It is traditionally used to treat gastrointestinal disorders. The objective of this study was to evaluate the role of ginger in management of nausea among patients receiving chemotherapy.

**Methods:**

This crossover interventional study was conducted to evaluate the role of ginger in management of nausea among patients receiving chemotherapy. Study was carried out at chemotherapy daycare of Shifa International Hospital Islamabad, with a sample size of 90 patients, using non-probability convenient sampling. Patients undergoing chemotherapy were given the dose of ginger (550 mg twice a day) for five consecutive days. On the next chemo cycle the same patients were given the placebo capsules of same color and weight for two times a day for five days. Patients and attendants were contacted for five days and being asked about post-chemotherapy nausea. Nausea and vomiting were measured by Rhodes scale.

**Results:**

Results showed that Rhodes score of patients taking Ginger capsules was significantly lower than those taking placebo (p-value < 0.05) in all the five days.

**Conclusion:**

Ginger significantly managed the nausea among patients receiving chemotherapy. Its natural antiemetic properties provide a convenient and safe way to reduce the post-chemotherapy nausea and vomiting.

## INTRODUCTION

Cancer is the uncontrolled spread and growth of cell. It has tendency to penetrate and proliferate among the body’s organs. Reducing risk factors like heavy alcohol use, tobacco use, and exposure to toxic chemicals can help prevent many types of cancer. However, numerous types of carcinomas are treated with radiation, surgery, and chemotherapy.^1^

Cancer management is a global health problem including Pakistan.^2^ Chemotherapy is a cancer treatment in which drugs are used to eradicate cancer cells. The mechanism of the chemotherapy is to stop or slow growth rate of cancer cells.^3^ Several adverse effects, including fatigue, weight loss, constipation, and diarrhea, can occur in cancer patients receiving chemotherapy; however, nausea is one of the most frequent side effects. It’s an unpleasant, subjective feeling that requires the patient to throw up.^4^

The aim of chemotherapy treatment is to cure the cancer, lower the risk of recurrence of cancer, relieve symptoms and to find out the better results in treatment of cancers.^5^ Chemotherapeutic drugs are given to the patients on the basis of required benefits and possible risks. Chemotherapy can have both long term and short term side effects. Short term adverse effects include nausea, vomiting, fatigue, hair loss and loss of appetite. Whereas, long term adverse effects include risk of secondary carcinoma, cognitive changes and neuropathy. These side effects vary from patient to patient and drug to drug.^6^

Among all the side effects nausea and vomiting are most commonly experienced adverse effects by patients receiving chemotherapy.^7^ This can also increase the patient’s anxiety toward the chemotherapeutic treatment. Nausea and vomiting related to chemotherapy is known as chemotherapy induced nausea and vomiting (CINV).^8^ CINV divided into three types: acute CINV which remains for the first day post chemotherapy; delayed CINV which begins after the first day post chemotherapy, this may last for a week; the last type is anticipatory CINV which patients experience with the drug agent used in the last chemo-cycle.^9^ Regardless of the extensive use of anti-emetic drugs, nausea is persistently reported in 70% of patients who were receiving chemotherapy.^10^ Ginger supplementation of 0.5 g–1.0 g on daily basis significantly helped in decline of the severity of acute chemotherapy-induced nausea in cancer patients.^11^

Ginger is given to the patients facing nausea and vomiting related to pregnancy in fact ginger also has a very productive role in management of vomiting and nausea after operation.^12^ Ginger root powder has convincingly decreased intensity of delayed and acute CINV in combination with other anti-emetic drugs.^9^

The severity of nausea and vomiting depends on the agent present in the chemotherapy drug and on the type of carcinoma patient is diagnosed with. The activation of 5-HT3 receptor plays a pivotal role in inducing nausea and vomiting. When a patient receives chemotherapy these receptors which are present in the central nervous system and gastrointestinal tract got triggered. The 5-HT3 receptor antagonists should be included as approved antiemetic therapy for patients who are at risk of facing CINV.^13^

Ginger *(Zingiber officinale)* is traditionally used to treat heart burn, nausea and vomiting. Ginger has capability of increasing the gut mobility and also increases bowel contractions.^14^ This impact of ginger is due to the antagonist effect of ginger on 5-HT3 receptors.^15^,^16^

## METHODS

It was a crossover interventional study design. This study was performed in chemotherapy daycare of Shifa International Hospital Islamabad which is a JCIA (joint commission international accredited) hospital. In the beginning as per sample size, 90 patients were enrolled for the trials but as the study progressed some of the patients left the study, few of them died and few did not continue the treatment in Shifa International Hospital. At the end of this study the number of remaining patients were 70. Sample size was calculated by using WHO calculator using parameters i.e. level of significance, confidence interval, expected prevalence and power of test.

### Ethical Approval:

Before implementation of study this project was presented and approved from research department (IRB) of Shifa International Hospital (IRB# 900-175-2017).

The inclusion criteria for this study involved patients undergoing chemotherapy with emetogenic potential scores ranging from two to five. These emetogenic potential scores were determined by the National Comprehensive Cancer Network (NCCN), which categorizes chemotherapy drugs on a scale from one (least emetogenic) to five (highly emetogenic). The study’s exclusion criteria comprised patients who had completed more than five cycles of chemotherapy, those with a known allergy to ginger or ginger products, participants with gastrointestinal (GI) cancer or any GI disorder, patients under the age of 16 years, patients with carcinoid tumor, mental disorders or a history of cranial surgery.

### Intervention:

In the current study, the selected patients were given with ginger capsules. Prior to administering the capsule, informed consent was obtained from every participant. Each capsule contained 550 mg of organic ginger powder. Patients undergoing chemotherapy received a dose of ginger (550 mg twice a day) for five consecutive days. During the next chemo cycle the same patients received the placebo dosage two times a day for five consecutive days. Patients follow up occurred at home, their contact numbers were taken and also involved patient’s attendants. Notably, the color and weight of capsules (ginger and placebo) were same and they were made in pharmacy of Shifa international hospital Islamabad.

**Fig.1:**
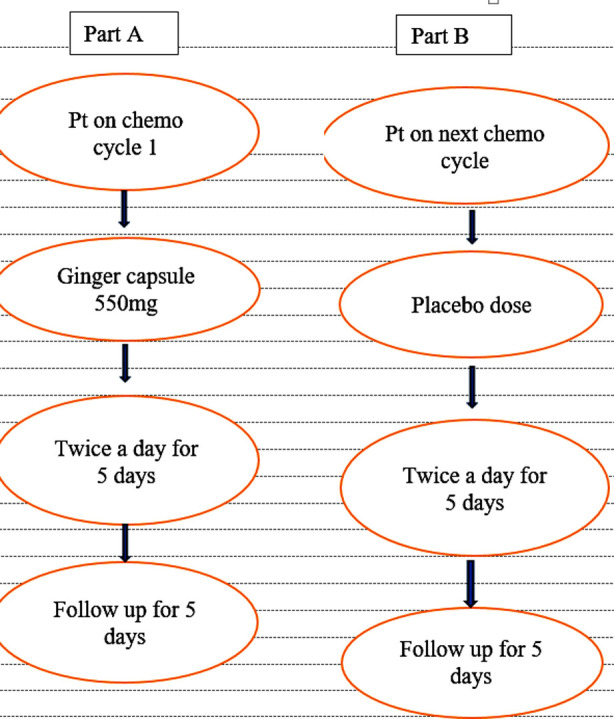
Study Flow Chart.

The data processed for the results consisted of 70 patients. The data of the remaining 20 patients was excluded as this could decrease the validity of the results. The current study was divided into two parts. In part one (Part-A), the patients received ginger capsules of 550 mg twice a day for five consecutive days and were followed at home to check the frequency of nausea and vomiting by using the Rhodes Scale index. In the second part (Part-B), the same patients on their next chemo cycle received placebo capsules of the same weight and color. Patients were followed at home, just like in Part-A. Both patients and the researcher were not aware of the ginger and placebo dosages. The double blinding was done to prevent bias. The study flow chart can be seen in Fig.1.

### Data collection tools:

In the current study, the Rhodes scale was used to measure the prevalence, severity, and score of nausea. Patients were followed at home, vomiting and nausea were evaluated using Rhodes index scoring.^9^ The Rhodes Scale is a questionnaire that can be administered once or twice a day. With the help of scoring criteria, a patient can report the frequency and changes in nausea and vomiting. This scale consists of eight questions with different scores. In this study reversed scoring of questions one, three, six and seven was done. In this study, scores were classified into no, mild, moderate, and severe, with 0 being the lowest and four being the highest. It means that as the score increases, the patient complains more about nausea. The validity of the tool was assessed by five panelist’s experts from the Oncology department, including two consultants, two nurses, and one pharmacologist. The content validity index (CVI) and Cronbach’s alpha coefficient values for the tool were 0.99 and 0.87, respectively.

## RESULTS

There was a total of 70 participants who completely participated in the current study. Among all the participants, the minimum age was 19 years, and the maximum age was 74 years. The mean age was 48.9 years, and the standard deviation (SD) was 13.27. Chemo-cycles were ranged from cycle 1-5. The mean and standard deviation of chemo cycles were 2.43 and 0.962, respectively. There were more female patients than male patients overall in the study, with 48 (68%) female patients and 22 (32%) male patients. As far as the type of carcinoma among patients was concerned, breast cancer was the top among all types of cancer. There were 35 (50%) females diagnosed with breast cancer (Table-I). The alarming sign of this study was that the patients who were coming with breast carcinoma were not only of old age, but there were also many who came with the diagnosis of breast carcinoma at a young age.

**Table-I:** Cancer Type with Gender.

Cancer Type	Gender		Total

	Male	Female	
Breast	-	35 (50.00%)	35 (50.00%)
Brain	-	01 (1.50%)	01 (1.50%)
DLBCL	-	01 (1.50%)	01(1.50%)
IRC	-	01 (1.50%)	01 (1.50%)
Leukemia	-	01 (1.50%)	01 (1.50%)
Liver	-	01(1.50%)	01 (1.50%)
Lung	10 (14.28%)	-	10 (14.28%)
Lymphoma	02 (3.00%)	-	02 (3.00%)
Myeloma	02 (3.00%)	01 (1.50%)	03 (4.50%)
Nasal	01 (1.50%)	-	01 (1.50%)
Ovarian	-	05 (7.50%)	05 (7.50%)
Pancreas	01 (1.50%)	-	01 (1.50%)
Prostate	03 (4.50%)	-	03 (4.50%)
Seg Cell	01 (1.50%)	-	01 (1.50%)
Throat	02 (3.00%)	-	02 (3.00%)
Thyroid	01 (1.50%)	-	01 (1.50%)
Uterus	-	01 (1.50%)	01 (1.50%)

### Inferential Statistics:

In the current study, data was normally distributed, the control and ginger (interventional) groups were the same patients receiving both a placebo and a ginger dosage; hence, the paired t test was applied to compare the mean Rhodes score of control and ginger groups. The mean Rhodes score of the control and ginger groups was from day one to day five is mentioned in Table-II.

**Table-II:** Paired Sample T Test to compare mean Rhodes Score of Control and Ginger Group.

Duration (Days)	Rhodes score (Mean ± SD)		P-value

	Ginger	Control	
01	1.81± 0.731	2.02 ± 0.78	0.035
02	1.75 ± 0.76	1.97± 0.77	0.030
03	1.60 ± 0.73	1.78 ± 0.73	0.073
04	1.48 ± 0.60	1.68 ± 0.72	0.008
05	1.49 ± 0.67	1.64 ± 0.70	0.050

On day one, the mean Rhodes scale of the ginger group was 1.81 ± 0.731, compared to the control group 2.02 ± 0.781. On day two, the mean Rhodes scale of the ginger group was 1.748 ± 0.765 as matched to the control group 1.966 ± 0.765. On day three the mean Rhodes scale of ginger group was 1.601 ± 0.733 as compared with the control group1.776 ± 0.733. On day four the mean Rhodes scale of ginger group was 1.484 ± 0.600 as compared with the control group 1.682 ± 0.722. On day five the mean Rhodes scale of ginger group was 1.487 ±0.673 as compared with the control group 1.645 ± 0.698 (Table-III).

**Table-III:** Paired sample T test.

Ginger and control comparison		Mean	SD	Std error mean
Pair 1	Day 1 control	2.0221	0.7805	0.95
	Day 1 Ginger	1.8051	0.7313	0.89
Pair 2	Day 2 control	1.9669	0.7710	0.94
	Day 2 Ginger	1.7482	0.7652	0.93
Pair 3	Day 3 control	1.7757	0.7335	0.89
	Day 3 Ginger	1.6011	0.7331	0.88
Pair 4	Day 4 control	1.6820	0.7229	0.88
	Day 4 Ginger	1.4835	0.6008	0.73
Pair 5	Day 5 control	1.6452	0.6984	0.85
	Day 5 Ginger	1.4871	0.6727	0.82

On day one the ginger group has significantly lower Rhodes scale score than control group, Mean difference was 0.22 ± 0.83 with P-value of 0.035. On day two the ginger group significantly lower Rhodes scale score than control group, mean difference is 0.22 ± 0.81 with P-value of 0.030. On day three the ginger group did not significantly lower Rhodes scale score than control group, mean difference is 0.17 ± 0.79 with P-value of 0.073. On day four the ginger group significantly lower Rhodes scale score than control group, mean difference is 0.20 ± 0.60 with P-value of 0.008. On day five the ginger group significantly lower Rhodes scale score than control group, mean difference is 0.16 ± 0.65 with P-value of 0.050.

## DISCUSSION

The current study was conducted to determine the effectiveness of ginger in the management of nausea among patients receiving chemotherapy. This study shows that the mean age of the patients was 48.95 years, which indicates that cancer is common in people over the age of 45. Furthermore, it was also confirmed from the literature that the mean age of cancer patients was 46 in a study conducted in Lahore to see the socioeconomic status of patients with breast cancer.^17^ In the present study, 68.12% patients were female, and out of them, 50.73% were diagnosed with breast cancer. It indicates that women are affected by breast cancer more commonly in Pakistan.

A similar study was held to find out the prevalence of breast cancer in Pakistan, and that study concluded that one in nine females is diagnosed with breast carcinoma. The incidence is increasing 2.5 times higher than in our neighboring countries i.e., Iran and India.^18^ In the current study, lung carcinoma was prevalent in males; out of 31.9% of males, 14.50% of patients were diagnosed with lung cancer. A study suggested that the lung cancer in Pakistan is more common in males because the highest prevalence rate of smoking among males is generally in East and South Asia.^19^ The present study has shown that ginger has significantly reduced nausea among patients receiving chemotherapy.

Another study has concluded that ginger has an effective impact on the management of nausea among patients receiving chemotherapy. Ginger helps to reduce emesis by acting on *5-HT3* receptors. Ginger is a *5-HT3* antagonist in nature, which reduces nausea.^9^ Another study by Sontakke, Thawani^20^, which was a RCT based trial and a double-blind designed study, had the main purpose of determining the effect of ginger compared to ondansetron and metoclopramide in 60 patients treated with cyclophosphamide of low dosage combined with other chemotherapeutic ingredients having the ability to cause mild nausea and vomiting.

The outcome shows the efficiency of ginger in managing nausea and vomiting, due to its antiemetic capability, which was equal to metoclopramide but lower than ondansetron. This was not analogous to the existing effort, because they gave ginger in place of antiemetic medications such as 5-HT3 antagonist instead of the standard antiemetic therapy such as a *5-HT3* antagonist. However, in the present trial, ginger was co-administered with the standard antiemetic regimens such as *dexamethasone*. Moreover, this work failed to examine the effectiveness of ginger on the occurrence and intensity of delayed CINV.

### Limitations:

The current study showed that patients undergoing chemotherapy who received ginger capsules experienced a significant reduction in nausea, which is strong evidence that ginger can manage post-chemo nausea and vomiting. The study’s small sample size and single study setting may limit the generalizability of the results, as the focus was solely on chemotherapy patients.

## CONCLUSION

Administering ginger alongside chemotherapy regimens can significantly reduce the nausea and vomiting. Its natural antiemetic qualities provide a convenient way to reduce the post- chemotherapy nausea and vomiting. Combating this debilitating symptom can help patient during the treatment with better outcome.

### Recommendations:

Ginger can be a better non-pharmacological choice in the management of chemotherapy-induced vomiting and nausea. Additionally, further researches like randomized control trials are needed to explore ginger’s efficacy in managing nausea among patients receiving chemotherapy with different types of cancers.

### Consent to participants:

An informed consent form was signed prior to data collection by all participants.

### Consent for publication:

I hereby grant permission for the publication of my research titled “Role of ginger in management of nausea among patients receiving chemotherapy”.

## Data availability statement:

The data that support the findings of this study are available on request from the corresponding author. The data are not publicly available due to restrictions, e.g., information that could compromise the privacy of research participants.

### Authors Contributions:

**ZA** contributed to conception, design and data acquisition and are responsible, accountable for the accuracy and integrity of the work.

**AF and MS** contributed to supervision, validation and interpretation equally.

**RI** contributed to drafting of article.

All authors have read, reviewed and approved the final manuscript.
